# Prevalence and Determinants of Mental Health among COPD Patients in a Population-Based Sample in Spain

**DOI:** 10.3390/jcm10132786

**Published:** 2021-06-24

**Authors:** Marta Fuentes-Alonso, Marta Lopez-Herranz, Ana López-de-Andrés, Zichen Ji, Rodrigo Jiménez-García, Clara Maestre-Miquel, José J. Zamorano-León, Isabel Jimenez-Trujillo, Javier de Miguel-Diez

**Affiliations:** 1 Respiratory Care Department, Hospital General Universitario Gregorio Marañón, Universidad Complutense de Madrid, Instituto de Investigación Sanitaria Gregorio Marañón (IiSGM), 28040 Madrid, Spain; mfuentesa@salud.madrid.org (M.F.-A.); jizich72@gmail.com (Z.J.); javier.miguel@salud.madrid.org (J.d.M.-D.); 2 Faculty of Nursing, Physiotherapy and Podology, Universidad Complutense de Madrid, 28040 Madrid, Spain; 3 Department of Public Health & Maternal and Child Health, Faculty of Medicine, Universidad Complutense de Madrid, 28040 Madrid, Spain; anailo04@ucm.es (A.L.-d.-A.); rodrijim@ucm.es (R.J.-G.); josejzam@ucm.es (J.J.Z.-L.); 4 School of Health Sciences, Universidad de Castilla la Mancha, 45600 Talavera de la Reina, Spain; Clara.Maestre@uclm.es; 5 Preventive Medicine and Public Health Teaching and Research Unit, Department of Medical Specialties and Public Health, Universidad Rey Juan Carlos, Alcorcón, 28922 Madrid, Spain; isabel.jimenez@urjc.es

**Keywords:** COPD, mental disorders, psychological distress, psychiatric medication consumption, national health survey, Spain

## Abstract

(1) Background: To assess the prevalence of mental disorders (depression and anxiety), psychological distress, and psychiatric medications consumption among persons suffering from COPD; to compare this prevalence with non-COPD controls and to identify which variables are associated with worse mental health. (2) Methods: This is an epidemiological case-control study. The data were obtained from the Spanish National Health Survey 2017. Subjects were classified as COPD if they reported suffering from COPD and the diagnosis of this condition had been confirmed by a physician. For each case, we selected a non-COPD control matched by sex, age, and province of residence. Conditional logistic regression was used for multivariable analysis. (3) Results: The prevalence of mental disorders (33.9% vs. 17.1%; *p* < 0.001), psychological distress (35.4% vs. 18.2%; *p* < 0.001), and psychiatric medications consumption (34.1% vs. 21.9%; *p* < 0.001) was higher among COPD cases compared with non-COPD controls. After controlling for possible confounding variables, such as comorbid conditions and lifestyles, using multivariable regression, the probability of reporting mental disorders (OR 1.41; 95% CI 1.10–1.82).), psychological distress (OR 1.48; 95% CI 1.12–1.91), and psychiatric medications consumption (OR 1.38 95% CI 1.11–1.71) remained associated with COPD. Among COPD cases, being a woman, poor self-perceived health, more use of health services, and active smoking increased the probability of suffering from mental disorders, psychological distress, and psychiatric medication use. Stroke and chronic pain were the comorbidities more strongly associated with these mental health variables. (4) Conclusions: COPD patients have worse mental health and higher psychological distress and consume more psychiatric medications than non-COPD matched controls. Variables associated with poorer mental health included being a woman, poor self-perceived health, use of health services, and active smoking.

## 1. Introduction

Chronic obstructive pulmonary disease (COPD) is a highly prevalent condition associated with high morbidity and mortality throughout the world [1]. Patients with this disease show a progressive deterioration of their respiratory functional capacity, which negatively affects their quality of life [2,3,4].

COPD patients present more frequently than the general population other concomitant comorbidities. These include cardiovascular disease (ischemic heart disease, heart failure, or stroke), high blood pressure, diabetes mellitus, kidney failure, osteoporosis, skeletal muscle dysfunction, lung cancer, and psychiatric diseases (depression and anxiety). These concomitant chronic diseases must be actively sought, since they interact with each other, making their diagnosis and treatment difficult, and worsen the prognosis [5]. Furthermore, they must be treated early, as they independently increase hospitalizations and mortality in COPD patients [6].

The prevalence of psychiatric disorders, including anxiety and depression, in COPD patients is higher than in the general population [7]. In addition, these pathologies can go unnoticed since, in many cases, it is difficult to distinguish between anxiety and the dyspnoea typical of COPD, due to the fact that both entities frequently overlap [8].

Anxiety is one of the known comorbidities with the highest risk of death, especially among women with a previous diagnosis of COPD, so it requires early care, since it is a potentially treatable condition [9]. Regarding depression, its prevalence in COPD patients is high, especially among those who are on a home oxygen therapy program, in whom it can reach 60% [10]. These patients have reduced physical activity and quality of life, also frequently presenting poor adherence to medical treatment.

Several negative consequences have been associated with untreated depression and anxiety in COPD patients [11]. Previous studies have shown that COPD patients with comorbid depression and anxiety are more likely to have exacerbations and hospital admissions. They also have greater symptom burden, higher disability, and poorer social functioning than patients with COPD alone [11,12,13]. In addition, evidence suggests that depression may be a risk factor for mortality among COPD patients [13].

Other studies have also shown the relevance of the coexistence of COPD and psychological distress. This mental alteration can impair the patient’s ability to cope with physical symptoms, leading to greater weakness and further worsening his psychological situation [14].

Furthermore, the effect of treating mental disorders on the optimal management of COPD patients is unclear. However, some authors have suggested that there is a positive influence of the consumption of psychiatric medication on COPD, since it would favor the use and adherence to maintenance inhalers, thus achieving greater control of the disease [15].

National surveys, such as the Spanish National Health Survey (SNHS), constitute the main source of information on the perceived health of the population. They represent a basic instrument for the knowledge of the health of the population, also providing useful data for health resource planning and research [16].

The aims of the current investigation, using the Spanish National Health Survey 2017 (SNHIS2017), were: (a) to assess the prevalence of self-reported mental disorders, psychological distress, and self-reported psychiatric medications consumption among persons suffering from COPD; (b) to compare these prevalence with sex-and-age matched non-COPD controls; and (c) to identify which socio-demographic and health-related variables are associated with reporting mental disorders, psychological distress, and consumption of psychiatric medications among persons suffering from COPD.

## 2. Materials and Methods

### 2.1. Study Design and Study Population

We conducted an epidemiological case-control study. The data for our investigation were obtained from the SNHIS2017. Details on the methodology of the SNHSI2017 are described elsewhere [17,18].

The SNHIS2017 was designed to provide reliable estimates, at both national and regional levels, of the Spanish Population aged 15 year or over and included a total sample size of 23,089 participants. The information collection period was from October 2016 to October 2017. Briefly, the SNHS2017 uses a three-stage sampling, the first stage units being the census tracts, the second being the main family dwellings, and in the last stage, an adult (aged ≥ 15 years old) who was randomly selected (Kish method) within each household [18]. Computer-assisted personal interview is the method used to collect the information. Given the low prevalence of COPD in Spain among those aged under 35 years, we included only subjects aged ≥ 35 years in our investigation [19].

### 2.2. Case Control Design

Subjects were classified as COPD sufferers (cases) if they reported to be suffering from COPD and that the diagnosis of this condition had been confirmed by a physician. Subjects who answered “no” to the presence of COPD were included to create the control group.

For each case, we selected a non-COPD control matched by sex, age, and province of residence. If more than one control was available per case, the selection of the case was carried out randomly. 

Details in the questions used to create our study variables can be found in Table S1 and in the SNHIS2017 methodology and questionnaire [17,18].

### 2.3. Study Variables

We measured mental health using three dependent variables:

1. The self-reported presence of a “mental disorder” was defined if the person interviewed reported to suffer from depression and/or anxiety with these conditions being diagnosed by a medical doctor.

2. The presence of “psychological distress” was assessed using the 12-item General Health Questionnaire 12 (GHQ-12). The Spanish version of the GHQ12 has been validated and a cut-point of ≥3 is recommended to identify individuals with psychological distress in previous investigations [20,21,22,23].

3. The variable “consumption of psychiatric medications” was created using questions regarding the self-reported use of physician-prescribed medications in the last two weeks. We considered psychiatric medications any of the following “tranquilizers (anxiolytics)”, “sedatives (anxiolytics)”, “sleeping pills (anxiolytics)”, and “antidepressants”.

Independent variables included: (a) socio-demographic characteristics such as “sex”, “age”, “living with a couple”, “educational level”, and “social class”; (b) health-related variables such as “self-rated health” and use of health care services in the last year (“emergency services”, “hospital admission”, and “visit to psychologist”); (c) self-reported presence of medical doctor diagnosed concomitant chronic diseases (“hypertension”, “heart diseases”, “arthrosis”, “stroke”, “diabetes mellitus”, “malignant tumors”, “chronic pain” and “accident permanent injuries”); and (d) lifestyle-related variables (“obesity”, “alcohol consumption”, “current smoking habit”, and “physical inactivity). Description and categories for these variables are shown in Table S1. 

If the participant answered “Don’t know” or “Don’t want to answer”, the case and control were excluded for the analysis of that variable.

### 2.4. Statistical Analysis

The distribution according to the independent study variables was described and compared for COPD cases and matched non-COPD controls. The statistics used for description included absolute frequencies and proportions for prevalence. To compare prevalence between cases and controls, bivariate conditional logistic regression models were applied. According to the literature, this is a valid alternative to the Chi-square McNemar’s test [24].

To assess the association of study variables with the presence of mental disorders, psychological distress, and consumption of psychiatric medications among COPD cases, we constructed three unconditional logistic regressions following Hosmer et al.’s recommendation [25]. To do so, we included in the multivariable models all the independent variables with significant bivariate associations (*p* < 0.10), with the dependent variable, as well as those considered scientifically relevant in other investigations [9,14,23]. In order to fit the multivariable model, the importance of each variable was verified. This included the examination of the Wald statistic for each variable and a comparison of each estimated coefficient with the coefficient from the univariate model containing only that variable. Variables that did not contribute to the model based on these criteria were eliminated and a new model was fitted. The new model was compared to the previous model using the Likelihood Ratio test. Furthermore, estimated coefficients for the remaining variables were compared to those from the full model. This process of deleting, refitting, and verifying continued until all the important variables were included in the model. The “enter modeling” method of STATA 14.0 was used for variable selection. Once the final model was obtained, the collinearity between variables was assessed by the variance inflation factor, and the interactions in the model was analyzed.

The odds ratio (OR) and 95% confidence intervals (CI) were used to measure association.

### 2.5. Sensitivity Analysis

Even if we matched COPD cases and non-COPD controls by age, sex, and province of residence, the distribution of other study variables, such as comorbidities and lifestyles, remained significantly different. Therefore, the confounding effect of these unadjusted variables could cause a biased association of COPD with the mental health variables analyzed. To prevent this bias, we constructed three conditional multivariable logistic regression models using COPD as the dependent variable. This way, we can assess the probability of reporting mental disorders, psychological distress, and consumption of psychiatric medications after controlling the confounding effect of other comorbidities and lifestyles variables. These models were generated using the methods described above and, although matched variables (age and sex) could never enter directly into the model due to their differences being always zero, we included them in interactions with the difference variables [26]. 

STATA program (StataCorp LP, Lakeway DriveCollege Station, Texas, USA) was the software used for matching and analysis with statistical significance set at two-tailed *p* < 0.05.

### 2.6. Ethical Aspects

In accordance with the Spanish legislation, because we used a public access dataset with anonymous data, the approval of an ethics committee was not needed. The database can be freely downloaded by anyone from the Spanish Ministry of Health webpage [27].

## 3. Results

The total number of COPD cases that could be matched with a sex-age-province of residence control was 1034; this represents 92.9% of all patients with COPD included in the SNHS2017 (1.113). The mean age of the study population was 68.29 years (SD 13.58).

The overall prevalence of mental disorders (33.9 % vs. 17.1%; *p* < 0.001), psychological distress (35.4% vs. 18.2%; <0.001), and consumption of psychiatric medications (34.1% vs. 21.9%; <0.001) among COPD cases was higher than among matched non-COPD controls. 

The prevalence of the mental health variables according to socio-demographic variables among cases and controls is shown in Table S2.

As can be seen for the three variables analyzed, the prevalence among COPD cases is significantly higher in all the categories of most socio-demographic variables compared with non-COPD controls.

Regarding sex, the prevalence of mental disorders (44.1% vs. 23.0%; *p* < 0.001), psychological distress (41.1% vs. 29.1%; *p* < 0.001), and consumption of psychiatric medications (44.2% vs. 23.2; *p* < 0.001) was higher among women suffering from COPD than men with this condition. In addition, it is remarkable that the highest prevalence was found among those COPD cases with lower educational and social class levels for the three mental health variables.

The prevalence of mental disorder according to health-related variables can be seen in Table S3. COPD cases reported higher prevalence of mental disorders than non-COPD controls for all the categories of the variables shown in Table S3 with exceptions made for those who had visited a psychologist in previous year with figures of 79.1% and 74.5% (*p* = 0.535), respectively. According to the chronic conditions analyzed, the highest prevalence of mental disorders is found among COPD cases who suffered from concomitant stroke (49.2%), malignant tumors (47.5%), accident with permanent injuries (43.9%), chronic pain (42.8%), and diabetes mellitus (42.6%). 

Table 1 shows the results of the multivariable logistic regression to identify those variables independently associated with suffering mental disorders among COPD patients. Variables positively associate with reporting mental disorders included being women (OR 1.75; 95% CI 1.54–1.96), higher age (exception made for those aged 80 or over), not living with a couple, lower educational level, worse self-rated health, visited a psychologist in the previous year (OR 7.50; 95% CI 6.21–9.06), stroke, malignant tumors, chronic pain, accident with permanent injuries, obesity, current smoking habit, psychological distress (OR 10.35; 95% CI 9.15–11.69), and consumption of psychiatric medications (OR 2.80; 95% CI 2.48–3.15).

The results of the sensitivity analysis to assess the independent association of reporting mental disorders with COPD after conditional logistic regression are shown in Table S4. The OR obtained for “mental disorders” was 1.41 (95% CI 1.10–1.82). This means that, after adjusting by study variables, COPD cases reported a 41% higher prevalence of mental disorders than non-COPD controls.

The prevalence of psychological distress among COPD cases and controls according to health-related variables can be seen in Table S5. As reported for mental disorders, COPD cases show higher prevalence of psychological distress than non-COPD controls for most of the categories of the variables. No significant differences are found for the categories “Very good/good” self-reported health, those who visited to psychologist in the previous year, and those who reported suffering from stroke. Over half of COPD cases who reported concomitant stroke (53.6%) and accident with permanent injuries (52.6%) suffered from psychological distress (Table S5).

The variables independently associated with reporting psychological distress among COPD cases after multivariable analysis are shown in Table 2. Women with COPD had a higher risk of reporting psychological distress than men (OR 1.27; 95% CI 1.15–1.37) and the oldest age group (aged 80 years or over) had significantly lower probability (OR 0.57; 95% CI 0.48–0.68) than the youngest (aged 35–59 years). Other sociodemographic variables that increased the risk of psychological distress among COPD cases included not living with a couple and low social class.

Cases with “Fair, bad or very bad” self-rated health were classified as psychological distress sufferers almost three times more than those with a “Very good/good” self-rated health (OR 2.92; 95% CI 2.64–3.22). The use of any of the health care services in the previous year (emergency room, hospital, and psychologist) variables analyzed were associated to presenting psychological distress among COPD cases.

Of the chronic conditions studied, suffering from heart diseases, stroke, diabetes mellitus, malignant tumors, chronic pain, and an accident with permanent injuries were associated to psychological distress. 

Regarding lifestyle variables, current smoking habit and physical inactivity predicted more psychological distress. As expected, there were significant associations between psychological distress and mental disorders (OR 2.72; 95% CI 2.41–3.06) and consumption of psychiatric medications (OR 1.77; 95% CI 1.57–2.00).

As can be seen in Table S6, after the sensitivity analysis, the probability of reporting “Psychological distress” was 48% higher among COPD cases when compared with non-COPD controls (OR 1.48; 95% CI 1.12–1.91).

The prevalence of self-reported consumption of psychiatric medications according to study variables is shown in Table S7. The prevalence found is higher among COPD cases than COPD controls for most categories of health variables analyzed. The highest prevalence among COPD subjects is for those who visited a psychologist in the previous year (75.8%), those suffering from malignant tumors (51.4%), and those suffering from stroke (49.2%).

The results of the multivariable analysis to identify which variables are independently associated with self-reported consumption of psychiatric medications among COPD cases are shown in Table 3.

Being a woman (OR 1.30; 95% CI 1.15–1.45) and having older age are variables associated with higher consumption of psychiatric medications. Compared with the youngest age group, those aged 80 years or more had 6.13 times higher probability of consuming these medications and the odds ratio increased lineally with age. Those with the lowest educational level had higher use of psychiatric medications.

Regarding health-related variables, we observed that worse self-rated health and the use of health services in the previous year are risk factors for the consumption of these drugs. Moreover, the presence of arthrosis, stroke, chronic pain, and current smoking habit was significantly associated with the use of these medications.

Alcohol consumption in last 12 months, slightly but significantly, decreased the probability of reporting consumption of psychiatric medications (OR 0.87; 95% CI 0.77–0.97).

Finally, there was a positive association of psychological distress (OR 1.81; 95% CI 1.59–2.04) and mental disorders (OR 10.46; 95% CI 9.26–11.82) with psychiatric medication utilization among COPD cases.

As found for mental disorders and psychological distress (GHQ12 ≥ 3), after conditional logistic regression (Table S8), COPD was significantly associated with reporting consumption of psychiatric medications (OR 1.38; 95% CI 1.11–1.71).

## 4. Discussion

The present study demonstrates that COPD patients have higher mental morbidity than subjects without COPD matched by age, sex, and province. These differences are found for the three variables studied and are practically always significant after stratifying by sociodemographic variable and when related to the use of health services and health states. Furthermore, after conditional multivariable logistic regression analysis, all the mental health variables remained significantly associated with suffering from COPD.

The prevalence of mental disorders, psychological distress, and consumption of psychiatric medication in our study was significantly higher in COPD patients than in controls. Systematic reviews have estimated a prevalence of depressive symptoms in COPD patients of around 25% [28], while for generalized anxiety disorders, figures of around 16% have been described, with a frequency ranging between 6 and 33% [29]. 

Other authors have pointed out that psychological distress can indirectly cause a greater risk of COPD, both through smoking [30] and apathy, which would lead the patient to have lower levels of self-care and physical activity [31]. On the other hand, the consumption of psychiatric medication in COPD patients can improve the use and adherence to inhaler treatment. This could be due to the fact that, once the depressive symptoms improve with specific treatment, the patient adopts a healthier behavior, which includes the regular taking of COPD medications, hence the importance of improving the care of mental disorders in patients with chronic conditions, including respiratory diseases [14].

Regarding gender, our results show that female sex increases the risk for the three variables studied. Furthermore, the prevalence of mental disorders was higher among COPD patients who had lower educational levels and belonged to a lower social class. In previous studies, it has been already shown that the prevalence of symptoms of depression and anxiety is higher among women, and therefore also the consumption of psychiatric medication. Furthermore, gender may be a predictor of depression after the manifestation of a primary anxiety disorder [32]. Other authors have also suggested that women have higher rates of psychological distress, as well as less perceived control over symptoms and a greater degree of functional decline than men [9].

Our results show that patients living without a partner have an increased risk of suffering from mental disorders and psychological distress. In this sense, various studies have suggested that subjects with COPD less often have a partner and, if they do, value daily support in a less positive way and do not receive the necessary emotional support as often compared with individuals without COPD [33]. This lack of support could be explained by the fact that COPD subjects and their partners are very limited in their daily lives and often have different perceptions of the disease [34]. However, another study has shown the importance of partners of subjects with COPD in coping with the disease [35].

We also highlight in our study that self-perceived poor health in COPD patients is associated with the three mental health variables studied. These results are in line with previous studies in which it has been observed that the health status of COPD patients is worse in relation to individuals who do not suffer from this disease [36,37]. However, it is important to bear in mind that often the instruments used to assess health status, such as the COPD assessment test or the pain scale, focus largely on bodily sensations and limitations of daily life, paying less attention to emotional consequences [38].

In our study, the comorbidities that were most strongly associated with the three dependent variables evaluated were stroke and chronic pain. COPD patients are at increased risk of stroke as a result of common risk factors, including age, smoking, systemic inflammation, and coagulopathy caused by COPD [39,40]. In this regard, nearly 8% of COPD patients have been reported to have a history of stroke [41] and approximately 4% of all deaths in COPD patients are related to stroke-related incidents [42]. Regarding chronic pain, recent studies have placed it as a frequent symptom in these patients, also exerting a negative impact on the degree of dyspnea, physical activity, and quality of life [43]. One of the most common locations is low back pain [44], followed by neck, trunk, and extremities [45,46,47]. Among the triggers that would contribute to the persistence of this chronic pain, it is worth highlighting the mechanical limitations for the movement of the rib cage, musculoskeletal disorders, osteoporosis, compression fractures, and the side effects of prolonged use of steroids [48].

Among the lifestyles analyzed in our study, it should be noted that smoking increases the probability of the three dependent variables. On the other hand, alcohol consumption seems to reduce the use of psychotropic drugs. In relation to the consumption of these substances (tobacco and alcohol), previous studies have placed them as a means to minimize emotional distress in this profile of patients [49]. In addition, alcohol consumption could mask depressive symptoms, becoming an alternative to face this problem and thus justifying the reduction in the use of psychotropic drugs. Regarding physical activity, previous studies have already shown that its practice is generally associated with a higher degree of mental health [50], a slowdown in the loss of lung function, and a decrease in the number of exacerbations of the disease [12,51]. Probably, the explanation for the fact that being physically active reduces psychological distress is that physical activity improves heart function and peripheral muscle function, as well as increasing maximum oxygen consumption, among other effects, which would have a positive impact on the perception of dyspnea and consequently on the quality of life and well-being of COPD patients [52].

Depression and anxiety in COPD patients are associated with a disproportionate increase in health care utilization rates and costs. In our study, the use of health services (especially the visit to the psychologist) was associated with worse mental health for the three variables measured. A population-based study among people with chronic conditions (including COPD) showed that depression doubled the likelihood of using medical care, functional disability, and absenteeism from work [53]. Other studies in this topic suggest that depression in COPD leads to an increased risk of acute exacerbation and hospital admission [54,55,56]. In any case, evidence from systematic reviews indicates that the presence of mental disorders (including depression and anxiety) increases the costs of care for long-term conditions by at least 45%, after controlling for their severity for physical illness [57,58,59,60,61,62].

The main strength of this study is the use of a large sample, nationally representative, which allows us to evaluate the mental health of patients with COPD. This fact provides great external validity to our results, since we start with a sample size of more than 1000 subjects from the Spanish population with a diagnosis of COPD instead of limiting ourselves to a selected sample from a hospital/primary care center.

In our investigation, we used three variables to assess mental health that even if they are not independent, as all components are related to others, each one provides additional information that complements the others and allows us to provide a wider vision of the effect of COPD on mental health and to establish comparisons with populations without COPD or affected by other chronic conditions.

The GHQ-12 has been barely used among COPD patients. This is a powerful multifactor screening tool, useful to detect mental disorders (depression/anxiety), social dysfunction, and loss of confidence [63]. People with COPD experience distress as a consequence of their chronic condition, and identifying them early is important to complement the information provided by other objective variables such as the diagnosis of depression and/or anxiety, because it allows us to quantify a problem that may not have been detected by the healthcare system yet [33,34]. In addition, psychological distress has been proven to be useful as a measure of social support [63]. In fact, in our investigation, it was significantly associated with not living with a partner.

The analysis of psychiatric medication consumption provides additional information because the drugs analyzed include not only antidepressants and anxiolytic but also sleeping pills, providing information on sleeping disorders, which are not considered in the other two variables. This variable also provides information on access to the healthcare system, as we can use this variable to identify whether adults with mental disorders are being undertreated.

The use of a population survey allows us to analyze the effect of socio-demographic and lifestyle variables that are not usually collected in the medical records so we can identify those individuals with the highest risk of suffering from these conditions and provide useful information to health authorities to prioritize the prevention and promotion strategies to these subgroups.

Finally, the validity of population health surveys to investigate association of mental health conditions and COPD has been previously reported [64,65,66].

On the other hand, the matched case-control design is effective in avoiding confusion regarding the effect of age, sex, and province of residence. However, it is worth highlighting the existence of certain limitations that must be taken into account. Thus, the retrospective nature of this study limits the information on the exposure and is susceptible to bias. Furthermore, the only source for evaluating information on self-perceived health is the self-report, so an over- or under-reporting may appear based on recall or desirability biases. Finally, the analysis of smoking habit was limited to “current smoking habit”, which does not account for the interaction between former smoker status and COPD and comorbidities that are associated with mental health disorders. In our models, no interactions of smoker status with COPD and other comorbidities were found. However, there is prior evidence indicating that mental disorders are associated with current and former smoking and are also associated with the presence of smoking-related disease [67].

## 5. Conclusions

In summary, our results indicate that mental morbidity is clearly higher in subjects with COPD compared with those without this disease after matching by age, sex, and province. This association remains significant after controlling the effect of other comorbid conditions and lifestyles.

Among COPD cases, being a woman increases the risk for the three variables, as well as poor self-perceived health. Higher use of health services is associated with worse mental health with the three variables measured. Among the lifestyles, we highlight that smoking increases the probability of the three study variables, while being physically active reduces psychological distress, and alcohol consumption seems to reduce the intake of psychotropic drugs. It is important to provide tools for the patients that allow them to live with COPD, as well as to detect and treat associated mental disorders early, since these have a negative influence, not only on the quality of life, but also on the control of the disease.

## Figures and Tables

**Table 1:** Variables associated with the self-reported presence of mental disorders among persons suffering from COPD. Results of multivariable unconditional logistic regression analysis. Spanish National Health Survey 2017.

Health Variables	Categories	COPD	
		Odds Ratio	95% Confidence Interval
Sex	Men	1	
	Women	1.75	(1.54–1.96)
Age groups	35–59 years	1	
	60–69 years	1.25	(1.05–1.50)
	70–79 years	1.41	(1.17–1.70)
	80 or more	0.87	(0.69–1.10)
Living with a couple	Yes	1	
	No	1.40	(1.25–1.58)
Educational level	University	1	
	Secondary	1.29	(1.07–1.56)
	Primary	1.60	(1.31–2.02)
Self-rated health	Very good/good	1	
	Fair/poor/very poor	2.17	(1.91–1.33)
Visit psychologist in last year	No	1	
	Yes	7.50	(6.21–9.06)
Stroke	No	1	
	Yes	1.49	(1.06–2.09)
Malignant Tumors	No	1	
	Yes	1.28	(1.03–1.58)
Chronic pain	No	1	
	Yes	1.72	(1.52–1.95)
Accident with permanent injuries	No	1	
	Yes	1.33	(1.10–1.61)
Obesity	No	1	
	Yes	1.23	(1.07–1.41)
Current smoking habit	No	1	
	Yes	1.47	(1.29–1.68)
Psychological distress (GHQ12 ≥ 3)	No	1	
	Yes	10.35	(9.15–11.69)
Consumption of psychiatric medications	No	1	
	Yes	2.80	(2.48–3.15)

GHQ-12, 12-item General Health Questionnaire. COPD Chronic Obstructive Pulmonary Disease. No two way interactions showed a significant association.

**Table 2:** Variables associated with the presence of psychological distress (GHQ12 ≥ 3), among persons suffering from COPD. Results of multivariable unconditional logistic regression analysis. Spanish National Health Survey 2017.

Health Variables	Categories	COPD	
		Odds Ratio	95% Confidence Interval
Sex	Men	1	
	Women	1.27	(1.15–1.37)
Age groups	35–59 years	1	
	60–69 years	0.97	(0.86–1.10)
	70–79 years	0.82	(0.68–1.00)
	80 or more	0.57	(0.48–0.68)
Living with a couple	Yes	1	
	No	1.25	(1.14–1.36)
Social class	Upper	1	
	Middle	1.12	(0.97–1.27)
	Low	1.21	(1.06–1.39)
Self-rated health	Very good/good	1	
	Fair/poor/very poor	2.92	(2.64–3.22)
Emergency services in last year	No	1	
	Yes	1.31	(1.20–1.44)
Hospital admission in last year	No	1	
	Yes	1.26	(1.10–1.45)
Visit to psychologist in last year	No	1	
	Yes	1.98	(1.68–2.32)
Heart diseases	No	1	
	Yes	1.22	(1.05–1.40)
Stroke	No	1	
	Yes	1.45	(1.11–1.91)
Diabetes mellitus	No	1	
	Yes	1.15	(1.00–1.33)
Malignant tumors	No	1	
	Yes	1.31	(1.10–1.56)
Chronic pain	No	1	
	Yes	1.43	(1.30–1.57)
Accident with permanent injuries	No	1	
	Yes	1.63	(1.42–1.88)
Current smoking habit	No	1	
	Yes	1.13	(1.02–1.24)
Physical inactivity	No	1	
	Yes	1.47	(1.37–1.61)
Consumption of psychiatric medications	No	1	
	Yes	1.77	(1.57–2.00)
Mental disorders	No	1	
	Yes	2.72	(2.41–3.06)

COPD Chronic Obstructive Pulmonary Disease. No two-way interactions showed a significant association.

**Table 3:** Variables associated with consumption of psychiatric medications among persons suffering from COPD. Results of multivariable unconditional logistic regression analysis. Spanish National Health Survey 2017.

Health Variables	Categories	COPD	
		Odds Ratio	95% Confidence Interval
Sex	Men	1	
	Women	1.30	(1.15–1.45)
Age groups	35–59 years	1	
	60–69 years	2.31	(1.87–2.85)
	70–79 years	3.61	(2.92–4.46)
	80 or more	6.13	(4.79–7.83)
Educational level	University	1	
	Secondary	1.24	(0.99–1.53)
	Primary	1.23	(1.02–1.48)
Self-rated health	Very good/good	1	
	Fair/poor/very poor	1.87	(1.64–2.12)
Emergency services in last year	No	1	
	Yes	1.46	(1.30–1.65)
Hospital admission in last year	No	1	
	Yes	1.29	(1.09–1.52)
Visit to psychologist in last year	No	1	
	Yes	5.10	(4.21–6.17)
Arthrosis	No	1	
	Yes	1.39	(1.22–1.59)
Stroke	No	1	
	Yes	1.39	(1.02–1.89)
Chronic pain	No	1	
	Yes	1.42	(1.26–1.60)
Alcohol consumption in last 12 months	No	1	
	Yes	0.87	(0.77–0.97)
Current smoking habit	No	1	
	Yes	1.17	(1.02–1.34)
Psychological distress (GHQ12 ≥ 3)	No	1	
	Yes	1.81	(1.59–2.04)
Mental disorders	No	1	
	Yes	10.46	(9.26–11.82)

GHQ-12, 12-item General Health Questionnaire. COPD Chronic Obstructive Pulmonary Disease.

## Data Availability

This database can be downloaded freely and without cost from the website of the Ministry of Health, Social Services, and Equality (https://www.mscbs.gob.es/estadEstudios/estadisticas/encuestaNacional/encuesta2017.htm). In any case, we consider that all relevant data are within the paper.

## Supplementary Materials

The following are available online at https://www.mdpi.com/article/10.3390/jcm10132786/s1, Table S1. Definition of variables used in our investigation according to the questions included in the Spanish National Health Interview Survey 2017. Table S2. Prevalence of mental disorder, psychological distress (GHQ12 ≥ 3), and consumption of psychiatric medications according to socio-demographic variables among subject with COPD and matched non-COPD controls. Spanish National Health Survey 2017. Table S3. Prevalence of mental disorder according to health variables among subject with COPD and matched non-COPD controls. Spanish National Health Survey 2017. Table S4. Multivariable conditional logistic regression to assess the presence of mental disorders among persons with and without COPD (dependent variable). Spanish National Health Survey 2017. Table S5. Prevalence of psychological distress (GHQ12 ≥ 3), according to health variables among subject with COPD and matched non-COPD controls. Spanish National Health Survey 2017. Table S6. Multivariable conditional logistic regression to assess the presence of psychological distress (GHQ12 ≥ 3) among persons with and without COPD (dependent variable). Spanish National Health Survey 2017. Table S7. Prevalence of consumption of psychiatric medications according to health variables among subject with COPD and matched non-COPD controls. Spanish National Health Survey 2017. Table S8. Multivariable conditional logistic regression to assess the consumption of psychiatric medications among persons with and without COPD (dependent variable). Spanish National Health Survey 2017.
