# Supplementary material for: Prevalence and Determinants of Mental Health among COPD Patients in a Population-Based Sample in Spain
Source: J Clin Med. 2021 Jun 24;10(13):2786. doi: 10.3390/jcm10132786 (PMC8268632; doi:10.3390/jcm10132786)
Supplement: Supplementary file 1 [file jcm-10-02786-s001.zip › jcm-1230083-supplementary.pdf]

Table S1. Definition of variables used in our investigation according to the questions included in the Spanish National Health Interview Survey 2017.

**Questions**	**Description and answer**	**Variables**	**Categories**
12 items Global health questionnaire, (GHQ-12)[1, 2]*	The GHQ-12 consists of 12 items, each assessing the severity of a mental problem over the last few weeks using a 4-point Likert-type scale (from 0 to 3). Positive items were corrected from 0 (always) to 3 (never), and negative ones from 3 (always) to 0 (never). Scores on the GHQ-12 are obtained from a summation of responses to the 12 questions, with the first two response options scoring 0 and the last two scoring 1 (0-0-1-1).	Psychological distress GHQ12>3	Yes: GHQ12>3<br>No: GHQ12 ≤3
- Next I am going to read you a list of types of medications, please tell me which one or more of them have you taken in the last 2 weeks?<br>- Was it prescribed by the doctor?	A list of 23 medications are read the person interviewed. For those medications with an affirmative answer in the first question, the second are completed consecutively for each specific medication.<br>1. Tranquilizers sedatives or "sleeping pills",<br>2. Antidepressants	Consumption of psychiatric medications	Yes: Those who answered "yes" to both question for options 1 and/or 2<br>No: Answered "no" to one or both questions for options 1 and 2
Which is your sex?	Men<br>Women	Sex	Men<br>Women
How old are you?	Age in years	Age groups	35-59 years<br>60-69 years<br>70-79 years<br>80 or more
Of the following, which better describes your current situation regarding who you live with?	1. Living with my husband/wife.<br>2. Living with my couple<br>3. Not living with a couple	Living with a couple	Yes. Options 1 and 2<br>No: Option 3
What level of education have you completed?	1. Does not know how to read or write<br>2. Incomplete primary education<br>3. Complete primary education (<br>4. First stage of Secondary Education, with or without a qualification<br>5. Elementary Spanish Upper Secondary Education])<br>6. Upper secondary education<br>7. Intermediate vocational training or equivalent<br>8. Advanced vocational training or equivalent<br>9. University studies or equivalent<br>10. Over university (master, PhD….)	Educational level	Primary Options 1 to 3<br>Secondary Options 4 to 8<br>University Options 9 and 10
The social class categories have been taken from the proposal made by the Spanish Society of Epidemiology's (SEE) Working Group on Determinants, in which social class is assigned according to occupation[3]	National Classification of Occupations 2011 (CNO2011), [4]	Social Class	Upper<br>Meddle<br>Lower
In the past twelve month. How is your perception of your general health status?	1. Very good<br>2. Good,<br>3. Fair<br>4. Bad<br>5. Very bad.	Self-rated health	Very good/ good: Options 1 and 2<br>Fair/poor/very poor: Options 3 to5
In the last 12 months, have you had to use an emergency service for any problem or illness?	1. Yes<br>2. No	Emergency services in last year	1. Yes<br>2. No

In the last 12 months, have you had to be hospitalized for at least one night?	1. Yes<br>2. No	Hospital admission in last year	1. Yes<br>2. No
In the last 12 months, have you visited a psychologist?	1. Yes<br>2. No	Visit to psychologist in last year	1. Yes<br>2. No
1.Do you have or have you ever had any of the following diseases or heath conditions?<br>2. Have you suffered this disease/health condition in the past 12 months?<br>3. Was this disease/health condition been diagnosed by a doctor?	A card with a list of 32 conditions is shown to the person interviewed after the first question, and for those conditions reported by the participant, the second and third questions are completed consecutively for each specific condition.	• Chronic obstructive pulmonary disease<br>• Mental disorders (anxiety and/or depression)<br>• Hypertension<br>• Heart diseases (myocardial infarction, angina, coronary disease)<br>• Arthrosis,<br>• Stroke<br>• Diabetes mellitus<br>• Malignant tumours<br>• Chronic pain (neck pain and/or low back pain)<br>• Accident with permanent injuries	Yes: Answered affirmative to the three questions.<br>No: All the rest
1. Could you tell me how tall you are, approximately, without shoes?<br>2. Could you tell me your weight, approximately, without shoes and clothes?	Body mass index is calculated with the formulae:<br>Weight in Kg/ (Height in meters)^2	Obesity	Yes: If Body mass index is 30 or over.<br>No: Yes: If Body mass index is under 30
During the past 12 months, how often have you had alcoholic beverages of any kind (i.e. beer, wine, spirits, distilled and mixed drinks, or other alcoholic beverages)	1. Daily or almost daily<br>2. 5-6 days per week<br>3. 3-4 days per week<br>4. 1-2 days per week<br>5. 2-3 days in a month<br>6. Once a month<br>7. Less than once a month<br>8. Not in the last 12 months, have I stopped drinking<br>9. Never or just a few sips to taste it throughout life	Alcohol consumption in last 12 months	Yes. Options 1 to 6<br>No: Option 7 to 9
Could you tell me if you smoke?	1. Yes, I smoke daily<br>2. Yes, I smoke, but not daily<br>3. I don't currently smoke but have smoked before<br>4. I neither smoke nor have I ever smoked regularly ..	Current smoking habit	Yes. Options 1 and 2<br>No: Option 3 and 4
Which of these possibilities best describes how often you do some physical activity in your free time?	1. I don't exercise. I occupy my free time almost completely sedentary.<br>2. I do some occasional physical or sports activity.<br>3. I do physical activity several times a month<br>4. I do sports or physical training several times a week	Physical inactivity	Yes. Option 1<br>No: Option 2 to 4

[1] D. Goldberg, P. Williams, User's Guide to the General Health Questionnaire, NFER-Nelson, Berkshire, 1988.[2] Muñóz PE, et al. Adaptación española del General Health Questionnaire (G.H.Q.) de D. P. Goldberg (un método de identificación de casos psiquiátricos en la comunidad of D. P. Goldberg.*Arch Neurobiol (Madr)*. 1979;42(2):139-158. [3]Instituto Nacional de Estadistica. Encuesta Nacional de Salud 2017. [National Health Survey 2017]. Available at https://www.ine.es/daco/daco42/clasificaciones/cno11_notas.pdfhttps://www.ine.es/en/metodologia/t15/t153041917_en.pdf. Accessed 12 February 2021. [4]Instituto Nacional de Estadistica. National Classification of Occupations 2011 (CNO2011), Available at https://www.ine.es/daco/daco42/clasificaciones/cno11_notas.pdf Accessed 26 February 2021.

Table S2. Prevalence of mental disorder, psychological distress (GHQ12 ≥3) and consumption of psychiatric medications according to socio-demographic variables among subject with COPD and matched non-COPD controls. Spanish National Health Survey 2017.

Socio-demographic variables	Categories	Mental disorders					Psychological distress GHQ12>3					Consumption of psychiatric medications				
		COPD		Non-COPD		p	COPD		Non-COPD		P	COPD		Non-COPD		p
		N	%	N	%		N	%	N	%		N	%	N	%	
Total		378	33.9	190	17.1	<0.001	385	35.4	199	18.2	<0.001	380	34.1	234	21.9	<0.001
Sex	Men	124	23.0	51	9.5	<0.001	153	29.1	70	13.2	<0.001	125	23.2	74	13.8	<0.001
	Women	254	44.1	139	24.3	<0.001	232	41.1	129	22.9	<0.001	255	44.2	169	29.5	<0.001
Age groups	35-59 years	110	39.7	41	14.9	<0.001	110	40.0	46	16.7	<0.001	93	33.5	37	13.4	<0.001
	60-69 years	78	33.5	40	17.2	<0.001	65	28.4	36	15.6	0.001	76	32.6	51	21.9	0.009
	70-79 years	89	33.3	54	20.6	0.001	83	31.4	44	17.0	<0.001	96	36.0	68	26.0	0.013
	80 or more	85	32.8	50	19.3	<0.001	108	43.9	62	24.9	<0.001	108	41.7	84	32.4	0.029
Living with a couple	No	214	40.2	101	20.6	<0.001	203	40.0	111	22.9	<0.001	213	40.0	130	26.5	<0.001
	Yes	164	28.1	89	14.4	<0.001	182	31.3	88	14.4	<0.001	167	28.6	113	18.3	<0.001
Educational level	Primary	192	36.3	109	21.2	<0.001	200	38.6	112	22.4	<0.001	210	39.7	144	28.0	<0.001
	Secondary	130	31.4	55	13.3	<0.001	122	29.9	62	15.0	<0.001	116	28.0	65	15.7	<0.001
	University	28	25.5	19	13.2	0.013	34	31.8	14	9.8	<0.001	24	21.8	20	13.9	0.131
Social class	Upper	27	23.9	22	14.2	0.054	33	29.7	17	11.0	<0.001	22	19.5	29	18.7	0.876
	Middle	100	29.1	59	15.0	<0.001	98	29.4	69	17.8	<0.001	99	28.8	77	19.5	0.003
	Low	235	37.5	100	18.8	<0.001	241	39.2	106	20.2	<0.001	244	38.9	130	24.5	<0.001

COPD Chronic Obstructive Pulmonary Disease. P value for comparison of prevalence between COPD and matched non-COPD subjects using bivariate conditional logistic regression.

Table S3. Prevalence of mental disorder according to health variables among subject with COPD and matched non-COPD controls. Spanish National Health Survey 2017.

Health variables	Categories	COPD		Non-COPD		
		n	%	n	%	p
Self-rated health	Very good/ good	56	18.7	42	6.9	<0.001
	Fair/poor/very poor	322	39.5	148	29.8	<0.001
Emergency services in last year	Yes	195	38.5	76	23.4	<0.001
	No	183	30.1	114	14.5	<0.001
Hospital admission in last year	Yes	93	40.1	33	26.6	0.011
	No	285	32.3	157	15.9	<0.001
Visit to psychologist in last year	Yes	72	79.1	35	74.5	0.535
	No	305	29.8	155	14.6	<0.001
Hypertension	Yes	196	38.4	98	21.3	<0.001
	No	182	30.1	92	14.2	<0.001
Heart diseases	Yes	119	39.7	42	24.6	0.001
	No	259	31.8	148	15.8	<0.001
Arthrosis	Yes	230	42.0	109	26.8	<0.001
	No	148	26.1	81	11.5	<0.001
Stroke	Yes	30	49.2	11	26.2	0.025
	No	348	33.0	179	16.8	<0.001
Diabetes mellitus	Yes	110	42.6	34	19.1	<0.001
	No	268	31.3	156	16.8	<0.001
Malignant Tumors	Yes	67	47.5	18	23.4	<0.001
	No	311	31.9	172	16.7	<0.001
Chronic pain	Yes	286	42.8	119	28.1	<0.001
	No	92	20.6	71	10.3	<0.001
Accident with permanent injuries	Yes	69	43.9	27	29.0	0.022
	No	309	32.3	163	16.0	<0.001
Obesity	Yes	102	33.9	44	21.3	0.002
	No	248	33.1	128	15.7	<0.001
Alcohol consumption in last 12 months	Yes	178	27.7	83	12.8	<0.001
	No	200	42.4	107	23.3	<0.001
Current smoking habit	Yes	97	40.1	24	13.6	<0.001
	No	281	32.2	166	17.8	<0.001
Physical inactivity	Yes	212	38.6	97	22.3	<0.001
	No	166	29.3	93	13.8	<0.001

COPD Chronic Obstructive Pulmonary Disease. P value for comparison of prevalence between COPD and matched non-COPD subjects using bivariate conditional logistic regression.

Table S4. Multivariable conditional logistic regression to assess the presence of mental disorders among persons with and without COPD (Dependent variable). Spanish National Health Survey 2017.

**Health variables**	Categories		
		Odds ratio	95% confidence interval
Mental disorders	No	1	
	Yes	1.41	1.10-1.82
Self-rated health	Very good/ good	1	
	Fair/poor/very poor	2.54	2.03-3.18
Visit to emergency room	No	1	
	Yes	1.43	1.15-1.8
Chronic pain	No	1	
	Yes	1.67	1.35-2.08
Heart disease	No	1	
	Yes	1.38	1.06-1.79
Obesity	No	1	
	Yes	1.50	1.19-1.89
Diabetes	No	1	
	Yes	1.30	1.05-1.60
Physical inactivity	No	1	
	Yes	1.26	1.07-1.46
Current smoking habit	No	1	
	Yes	1. 48.	1.29-1.68

COPD Chronic Obstructive Pulmonary Disease. No two-way interactions showed a significant association.

Table S5. Prevalence of Psychological distress (GHQ12 ≥3), according to health variables among subject with COPD and matched non-COPD controls. Spanish National Health Survey 2017.

Health variables	Categories	COPD		Non-COPD		
		n	%	n	%	P
Self-rated health	Very good/ good	37	12.6	53	8.7	0.064
	Fair/poor/very poor	348	43.7	146	30.2	<0.001
Emergency services in last year	Yes	212	43.1	83	26.0	<0.001
	No	173	29.0	116	15.0	<0.001
Hospital admission in last year	Yes	111	49.8	35	28.9	<0.001
	No	274	31.6	164	16.9	<0.001
Visit to psychologist in last year	Yes	28	68.2	22	53.2	0.095
	No	325	32.5	174	16.6	<0.001
Hypertension	Yes	202	40.4	93	20.6	<0.001
	No	183	31.1	106	16.5	<0.001
Heart diseases	Yes	129	44.3	46	27.7	<0.001
	No	256	32.1	153	16.5	<0.001
Arthrosis	Yes	236	44.3	97	24.3	<0.001
	No	149	26.8	102	14.7	<0.001
Stroke	Yes	30	53.6	14	35.0	0.097
	No	355	34.4	185	17.6	<0.001
Diabetes mellitus	Yes	97	38.6	33	18.9	<0.001
	No	288	34.4	166	18.1	<0.001
Malignant Tumors	Yes	68	49.6	14	18.7	<0.001
	No	317	33.3	185	18.2	<0.001
Chronic pain	Yes	282	43.0	109	26.0	<0.001
	No	103	23.8	90	13.3	<0.001
Accident with permanent injuries	Yes	81	52.6	20	22.0	<0.001
	No	304	32.5	179	17.8	<0.001
Obesity	Yes	110	36.9	42	20.6	<0.001
	No	250	34.2	133	16.5	<0.001
Alcohol consumption in last 12 months	Yes	173	27.3	98	15.2	<0.001
	No	212	46.5	101	22.5	<0.001
Current smoking habit	Yes	88	37.1	24	13.7	<0.001
	No	297	34.9	175	19.0	<0.001
Physical inactivity	Yes	233	43.6	113	26.7	<0.001
	No	152	27.4	86	12.8	<0.001

COPD Chronic Obstructive Pulmonary Disease. P value for comparison of prevalence between COPD and matched non-COPD subjects using bivariate conditional logistic regression.

Table S6. Multivariable conditional logistic regression to assess the presence of Psychological distress (GHQ12 ≥3) among persons with and without COPD (Dependent variable). Spanish National Health Survey 2017.

**Health variables**	Categories	Odds ratio	95% confidence interval
Psychological distress (GHQ12 ≥3),	No	1	
	Yes	1.48	1.12-1.91
Self-rated health	Very good/ good	1	
	Fair/poor/very poor	2.50	1.99-3.14
Visit to emergency room	No	1	
	Yes	1.43	1.05-1.96
Chronic pain	No	1	
	Yes	1.69	1.36-2.10
Heart disease	No	1	
	Yes	1.41.	1.04-1.86
Obesity	No	1	
	Yes	1.49	1.19-1.89
Diabetes	No	1	
	Yes	1.28	1.04-1.57
Physical inactivity	No	1	
	Yes	1.29	1.09-1.50
Current smoking habit	No	1	
	Yes	1. 46.	1.28-1.66

COPD Chronic Obstructive Pulmonary Disease. No two-way interactions showed a significant association.

Table S7. Prevalence of consumption of psychiatric medications according to health variables among subject with COPD and matched non-COPD controls. Spanish National Health Survey 2017.

Health variables	Categories	COPD		Non-COPD		
		n	%	n	%	p
Self-rated health	Very good/ good	44	14.7	50	8.2	0.002
	Fair/poor/very poor	336	41.2	193	38.9	0.417
Emergency services in last year	Yes	188	37.0	98	30.2	0.042
	No	192	31.6	145	18.5	0.000
Hospital admission in last year	Yes	100	42.9	43	34.7	0.130
	No	280	31.7	200	20.3	0.000
Visit to psychologist in last year	Yes	69	75.8	32	68.1	0.331
	No	310	30.3	211	19.9	0.000
Hypertension	Yes	195	38.2	143	31.0	0.018
	No	185	30.5	100	15.4	0.000
Heart diseases	Yes	132	44.0	68	39.8	0.371
	No	248	30.4	175	18.7	0.000
Arthrosis	Yes	243	44.4	140	34.4	0.002
	No	137	24.1	103	14.7	0.000
Stroke	Yes	30	49.2	17	40.5	0.425
	No	350	33.2	226	21.2	0.000
Diabetes mellitus	Yes	109	42.2	52	29.2	0.006
	No	271	31.6	191	20.5	0.000
Malignant Tumors	Yes	74	51.4	21	27.3	0.001
	No	307	31.5	222	21.5	0.000
Chronic pain	Yes	294	43.9	147	34.8	0.003
	No	86	19.2	96	14.0	0.019
Accident with permanent injuries	Yes	66	42.0	27	29.0	0.043
	No	314	32.7	216	21.3	0.000
Obesity	Yes	98	32.5	53	25.6	0.097
	No	254	33.9	159	19.5	0.000
Alcohol consumption in last 12 months	Yes	177	27.5	110	16.9	0.000
	No	203	42.9	133	29.0	0.000
Current smoking habit	Yes	88	36.4	26	14.7	0.000
	No	292	33.4	217	23.3	0.000
Physical inactivity	Yes	218	39.6	126	29.0	0.000
	No	162	28.6	117	17.4	0.000
Mental disorders	Yes	253	66.9	142	74.7	0.057
	No	127	17.2	101	11.0	0.000
Psychological distress (GHQ12≥3)	Yes	210	54.5	101	50.8	0.384
	No	158	22.4	135	15.1	0.000

COPD Chronic Obstructive Pulmonary Disease. P value for comparison of prevalence between COPD and matched non-COPD subjects using bivariate conditional logistic regression.

Table S8. Multivariable conditional logistic regression to assess the consumption of psychiatric medications among persons with and without COPD (Dependent variable). Spanish National Health Survey 2017.

**Health variables**	Categories	Odds ratio	95% confidence interval
Consumption of psychiatric medications	No	1	
	Yes	1.38	1.11-1.71
Self-rated health	Very good/ good	1	
	Fair/poor/very poor	2.53	2.03-3.15
Visit to emergency room	No	1	
	Yes	1.52	1.23-1.87
Chronic pain	No	1	
	Yes	1.51	1.29-1.89
Heart disease	No	1	
	Yes	1.46	1.14-1.87
Obesity	No	1	
	Yes	1.46	1.17-1.82
Diabetes	No	1	
	Yes	1.34	1.07-1.69
Physical inactivity	No	1	
	Yes	1.19	1.03-1.41
Current smoking habit	No	1	
	Yes	1. 51.	1.30-1..73

COPD Chronic Obstructive Pulmonary Disease. No two-way interactions showed a significant association.
